# Parity-time-symmetric quantum critical phenomena

**DOI:** 10.1038/ncomms15791

**Published:** 2017-06-08

**Authors:** Yuto Ashida, Shunsuke Furukawa, Masahito Ueda

**Affiliations:** 1 Department of Physics, The University of Tokyo, 7-3-1 Hongo, Bunkyo-ku, Tokyo 113-0033, Japan; 2 RIKEN Center for Emergent Matter Science (CEMS), Wako, Saitama 351-0198, Japan

## Abstract

Synthetic non-conservative systems with parity-time (PT) symmetric gain–loss structures can exhibit unusual spontaneous symmetry breaking that accompanies spectral singularity. Recent studies on PT symmetry in optics and weakly interacting open quantum systems have revealed intriguing physical properties, yet many-body correlations still play no role. Here by extending the idea of PT symmetry to strongly correlated many-body systems, we report that a combination of spectral singularity and quantum criticality yields an exotic universality class which has no counterpart in known critical phenomena. Moreover, we find unconventional low-dimensional quantum criticality, where superfluid correlation is anomalously enhanced owing to non-monotonic renormalization group flows in a PT-symmetry-broken quantum critical phase, in stark contrast to the Berezinskii–Kosterlitz–Thouless paradigm. Our findings can be experimentally tested in ultracold atoms and predict critical phenomena beyond the Hermitian paradigm of quantum many-body physics.

## Abstract (web summary)

Parity-time (PT) symmetry has been mainly studied in optical and weakly interacting open quantum systems without many-body correlations. Here the authors show that in a strongly correlated many-body system the interplay between correlations and PT symmetry leads to the emergence of new critical phenomena.

Studies of phase transitions and critical behaviour in non-Hermitian systems date back to the discovery of the Lee-Yang edge singularity^1^, where an imaginary magnetic field in the high-temperature Ising model was demonstrated to trigger an exotic phase transition. More recently, the real-to-complex spectral phase transition has been found in a broad class of non-Hermitian Hamiltonians that satisfy parity-time (PT) symmetry^2^. While such systems were once considered to be of purely academic interest, related questions are now within experimental reach^3^,^4^,^5^,^6^,^7^.

A Hamiltonian 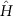 is said to be PT-symmetric if it commutes with the combined operator 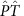, but not necessarily with 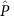 and 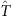 separately. Here 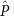 and 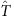 are the parity and time-reversal operators, respectively. PT symmetry is said to be unbroken if every eigenstate of 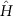 is PT-symmetric; then, the entire spectrum is real even though 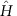 is not Hermitian. PT symmetry is said to be spontaneously broken if some eigenstates of 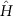 are not the eigenstates of the PT operator even though 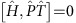; then, some pairs of eigenvalues of 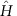 become complex conjugate to each other. PT symmetry breaking is typically accompanied by the coalescence of eigenstates and that of the corresponding eigenvalues at an exceptional point^8^ in the discrete spectrum or the spectral singularity^9^ in the continuum spectrum. While these features also hold for a certain class of antilinear symmetries^10^, PT symmetry allows experimental implementations by spatial engineering of gain–loss structures, leading to a rich interplay between theory and experiment in optics^4^,^5^,^6^,^7^,^11^, superconductors^12^, atomic physics^13^ and optomechanics^14^. In particular, the real-to-complex spectral transition (PT transition) has been observed in experiments of classical systems^15^. In all these developments, however, many-body correlations still play no role.

Quantum critical phenomena, in contrast, arise from collective behaviour of strongly correlated systems and exhibit universal long-distance properties. In view of recent developments in designing open many-body systems in ultracold atoms^16^,^17^,^18^,^19^,^20^ and exciton–polariton condensates^21^, it seems ripe to explore the role of PT symmetry in quantum critical phenomena and ask whether or not the concept of the universality need be extended in synthetic non-conservative systems.

Here we report that a combination of spectral singularity and quantum criticality yields an exotic critical point in the extended parameter space and that, in the PT-broken phase, a local gain–loss structure results in an anomalous enhancement of superfluid correlation owing to semicircular renormalization group (RG) flows. This contrasts sharply with the suppression of superfluid correlation due to hyperbolic RG flows in the Berezinskii–Kosterlitz–Thouless (BKT) paradigm. Our findings demonstrate that the interplay between many-body correlations and PT symmetry leads to the emergence of quantum critical phenomena beyond the Hermitian paradigm of quantum many-body physics.

## Results

### Parity-time-symmetric sine-Gordon model

We consider a class of one-dimensional (1D) quantum systems described by the field theory Hamiltonian


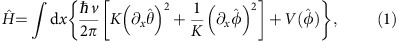


where 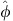 is a scalar field, 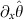 is its conjugate momentum satisfying 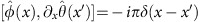, and 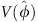 is a potential for the field 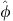. Without the potential term, equation (1) is known as the Tomonaga-Luttinger liquid (TLL) Hamiltonian, which gives a universal framework for describing 1D interacting bosons and fermions^22^. Here, *v* is the sound velocity, the TLL parameter *K* characterizes the interaction strength, and 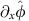 and 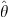 are related to the density and the Josephson phase, respectively. The introduction of the cosine potential 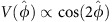 results in the sine-Gordon model, which describes the BKT transition to a gapped phase. For bosons on a lattice, this corresponds to a superfluid-to-Mott-insulator (MI) transition^23^. Here we consider a generalization to the PT-symmetric case by adding an imaginary contribution to the potential term as follows:


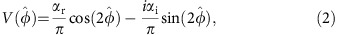


where *α*_r_ and *α*_i_ characterize the strengths of the real and imaginary parts of the potential. When the real part becomes relevant, it suppresses the fluctuations of 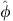, stabilizing a non-critical, gapped phase. In contrast, if the imaginary part is relevant, it facilitates the fluctuations of 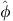 and enhances correlation in the conjugate field 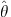, as we will see later. The field theory (1) with the potential (2) satisfies PT symmetry since the field 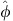 has odd parity. The PT-symmetric Hamiltonian 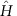 can be implemented by a continuously monitored 1D interacting ultracold atoms (see Supplementary Note 1 and Supplementary Fig. 1).

We note that if *α*_r_>*α*_i_, 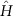 has a real spectrum and thus PT symmetry is unbroken. This can be proved by the theorem^24^ which states that the spectrum is real if and only if there exists an operator 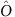 satisfying 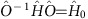, where 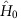 is a Hermitian operator. Indeed, we can explicitly construct such an operator for *α*_r_>*α*_i_ by the choice of 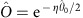, where 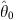 is a constant part of 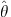 and *η* ≡ arctanh(*α*_i_/*α*_r_). Then, the potential term in the effective field theory is transformed to 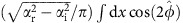 and 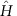 reduces to the sine-Gordon Hamiltonian^25^. Divergence of *η* at *α*_r_=*α*_i_ signals spontaneous breaking of PT symmetry.

### Renormalization group analysis

To unravel the universal critical behaviour of the PT-symmetric Hamiltonian 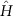, we perform an RG analysis^26^ to obtain the following set of flow equations which are valid up to the third order in *g*_r,i_:


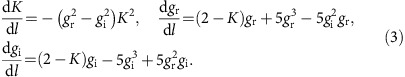


**Figure 1:**
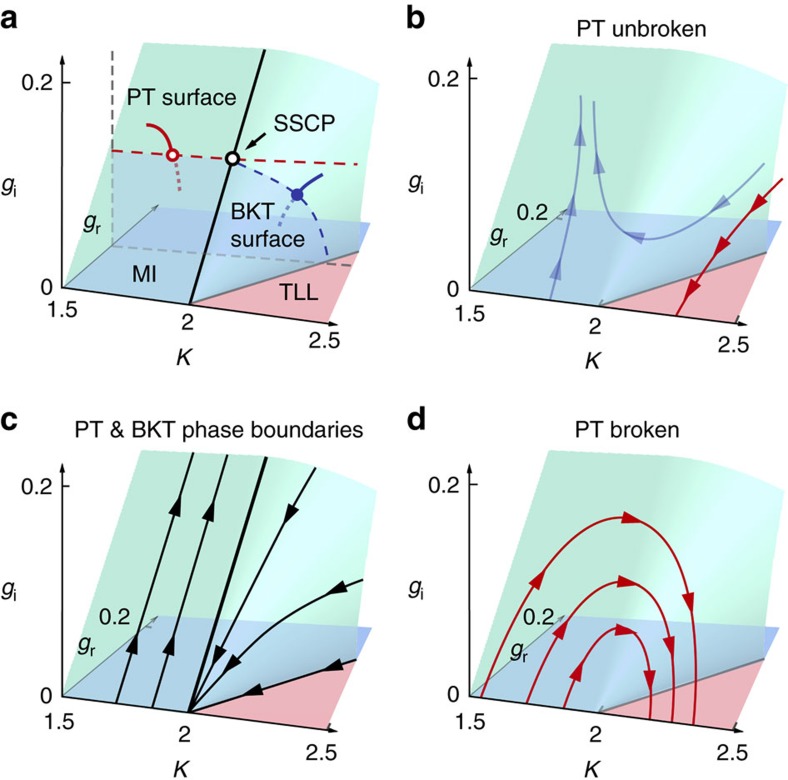
Quantum critical phenomena in PT-symmetric many-body systems. (**a**) 3D phase diagram of a PT-symmetric many-body system in the parameter space (*K*, *g*_r_, *g*_i_). Here *K* and *g*_r_ (*g*_i_) characterize the strength of the inter-particle interaction and the depth of the real (imaginary) part of a complex potential, respectively. The MI and TLL phases are separated by the surface of the BKT transition for *K*>2 and that of the PT transition for *K*<2. An example of the BKT (PT) transition is illustrated by the blue (red) curve with the transition point indicated by the filled (open) circle. The MI (TLL) phase corresponds to the 3D region containing the blue (red) shaded plane at *g*_i_=0. On the critical line with *K*=2 lies a SSCP (black open circle). Dashed lines indicate the phase boundaries on the plane with fixed *g*_r_ for comparison with numerical results in Fig. 2a. (**b**) Hyperbolic RG flows in a PT-unbroken region (*g*_i_<*g*_r_), which reproduce the conventional flow diagram in the sine-Gordon model. (**c**) RG flows on the two phase boundaries separated by an unconventional fixed line (thick black line). (**d**) Unconventional semicircular RG flows in a PT-broken region (*g*_i_>*g*_r_). Along each flow, the TLL parameter *K* monotonically increases, indicating the anomalous enhancement of the superfluid correlation.

Here *l* is the logarithmic RG scale and *g*_r,i_ ≡ *α*_r,i_*a*^2^/(*ħv*) are the dimensionless coupling constants with *a* being a short-distance cut-off. The velocity *v* stays constant to all orders in *g*_r,i_ because of the Lorentz invariance of the theory. In contrast to the two-dimensional phase diagram of the conventional sine-Gordon model, the PT-symmetric system has the three-dimensional (3D) phase diagram (Fig. 1a). When PT symmetry is unbroken, that is, *g*_i_<*g*_r_, the spectrum is equivalent to that of the closed system as discussed above and the conventional RG flow diagram with hyperbolic flows is reproduced (Fig. 1b). Here the BKT boundary between the superfluid TLL phase and the MI phase extends over the curved surface. We note that the operator 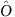 does not affect the critical properties of the ground state since it only modifies the zero modes associated with the field 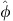. Since the non-Hermitian term can arise from the measurement backaction, the quantum phase transition induced by increasing *g*_i_ may be regarded as measurement-induced.

In the strongly correlated regime *K*<2, a new type of quantum phase transition appears on the PT threshold plane *g*_i_=*g*_r_. This phase transition is accompanied by spontaneous breaking of PT symmetry in eigenstates, contrary to the ordinary BKT transition exhibiting no symmetry breaking. The BKT and PT phase boundaries merge on the line defined by *K*=2 and *g*_i_=*g*_r_ (Fig. 1c). In general, at the PT symmetry-breaking point, the spectral singularity^9^ arises where two or more eigenvalues as well as their eigenstates coalesce in the continuum spectrum. In optics, the spectral singularity leads to unidirectional wave phenomena^5^. In contrast, in many-body systems, the coexistence of the spectral singularity and the quantum criticality at *g*_i_=*g*_r_ and *K*=2 results in what we term a spectral singular critical point, which represents a unique universality class in non-conservative systems.

When the PT symmetry is broken, that is, *g*_i_>*g*_r_, unconventional RG flows emerge: starting from the *K*<2 side, *g*_r,i_ and *K* initially increase, and after entering the *K*>2 side, the flow winds and converges to the fixed line with *g*_r,i_=0 (Fig. 1d). Physically, this significant increase in the TLL parameter *K* indicates that the superfluid correlation decays more slowly and is thus enhanced by the non-Hermiticity of an imaginary potential. The enhancement is viewed as anomalous because, in the conventional BKT paradigm, a real potential suppresses the fluctuation of 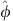 and stabilizes the gapped MI phase for *K*<2. Moreover, owing to the semicircular RG flows, the imaginary potential allows for a substantial increase of the TLL parameter *K* even if its strength *g*_i_ is initially very small. The PT-broken phase exhibits other observable consequences such as anomalous lasing and absorption as observed in optics^27^ (see Supplementary Note 2 for the experimental implementation in ultracold atoms).

### Ground-state phase diagram of the lattice model

**Figure 2:**
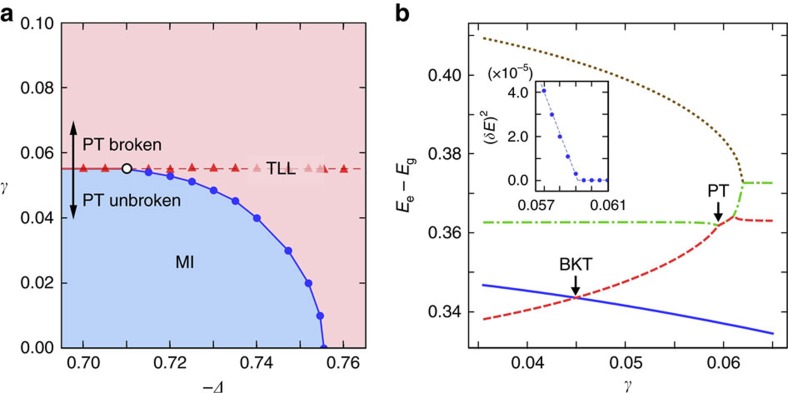
Phase diagram and finite-size spectrum. (**a**) Ground-state phase diagram of the PT-symmetric many-body lattice Hamiltonian. The MI and TLL phases are separated by the BKT transition (blue curve with filled circles) and PT-symmetry breaking (red line with filled triangles). The point where the two boundaries merge defines the SSCP (open circle). (**b**) Typical low-energy excitation spectrum in the lattice model. The three lowest levels in the *S*^*z*^=0 sector (red, green and yellow curves from the lowest), and the lowest excitation energy to the *S*^*z*^=±4 sector (blue curve) are plotted. Here 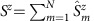 is the total magnetization. The energy difference *δE* between the two coalescing levels (for example, red and green) obeys the square-root scaling (inset) and closes at the PT-symmetry breaking point. The BKT transition point corresponds to a crossing of appropriate levels (red and blue). We set the parameter *h*_*s*_=0.1 for both (**a**,**b**). In (**a**), the plotted data are obtained through extrapolation to the thermodynamic limit, while the data in **b** are obtained for *N*=16 and −*Δ*=0.735. The plotted variables are dimensionless since we set *J*=1.

To numerically demonstrate these findings, we introduce a lattice Hamiltonian


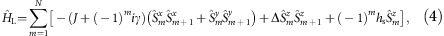


whose low-energy behaviour is described by the PT-symmetric effective field theory 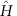. Here 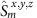 are the spin–1/2 operators at site *m* and the parameters (−Δ, *h*_s_, *γ*) are related to (*K*, *g*_r_, *g*_i_) in the field theory, where we set *J*=1. The non-Hermitian term represents a periodic gain–loss structure and effectively strengthens the amplitude of the hopping term, leading to enhanced superfluid correlation. The determined phase diagram and a typical exact finite-size spectrum are shown in Fig. 2. The BKT transition is identified as a crossing point of appropriate energy levels^28^ and the PT threshold is determined as a coalescence point in low-energy levels, as detailed in Methods section and Supplementary Methods. The coalescence point is found to be an exceptional point from the characteristic square-root scaling^8^ of the energy gap (see the inset figure in Fig. 2b). We note that, above the PT threshold, some highly excited states turn out to have positive imaginary parts of eigenvalues and cause the instability in the long-time limit. The presence of such high-energy unstable modes is reminiscent of parametric instabilities in exciton–polariton systems^29^, and can ultimately destroy the 1D coherence^30^. In our set-up, where the imaginary term is adiabatically ramped up, the amplitudes of these unstable modes can greatly be suppressed and the system can remain, with almost unit fidelity, in the ground state in which the critical behaviour is sustained (see Supplementary Note 3 and Supplementary Figs 2 and 3 for details).

### Numerical demonstration of enhanced superfluid correlation

**Figure 3:**
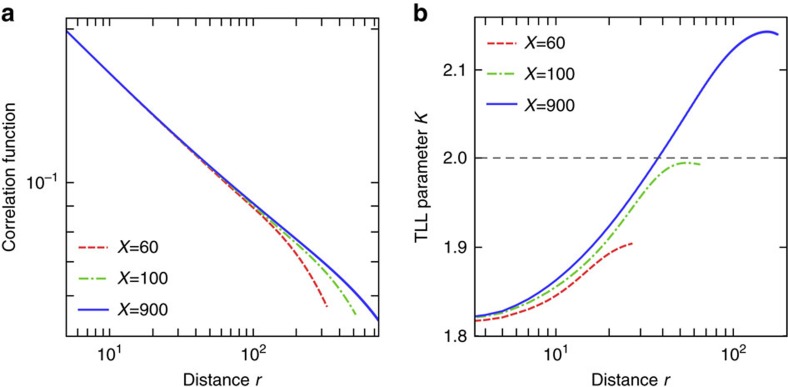
Anomalous enhancement of superfluid correlation in the PT-broken quantum critical phase. (**a**) Critical decay of the correlation function 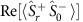. (**b**) TLL parameter *K* as a function of the distance *r*, giving the critical exponent of the correlation function, 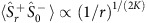. The exponent is extracted by the linear fitting of the correlation function in the log-log plot around the distance *r*. The parameters are set to be −*Δ*=0.61, *h*_*s*_=0.1 and *γ*=0.08, and *χ* denotes the dimension of the matrix product state that controls the accuracy of the iTEBD simulation.

To demonstrate the anomalous enhancement of superfluid correlation in the PT-broken regime, we have performed numerical simulations using the infinite time-evolving block decimation (iTEBD) algorithm^31^. The correlation function exhibits the critical decay with a varying critical exponent and the corresponding TLL parameter significantly increases, surpassing *K*=2 as shown in Fig. 3. Physically, this enhancement of superfluid correlation at long distances can be interpreted as follows. A local gain–loss structure introduced by the imaginary term causes locally equilibrated flows^15^ in the ground state. This results in the enhancement of fluctuations in the density, or equivalently, the suppression of fluctuations in the conjugate phase. It is this effect that increases the superfluid correlation. The numerical results are consistent with the analytical arguments given above, and demonstrate that the RG analysis is instrumental in studying critical properties of a non-Hermitian many-body system.

### Experimental realization in a one-dimensional Bose gas

**Figure 4:**
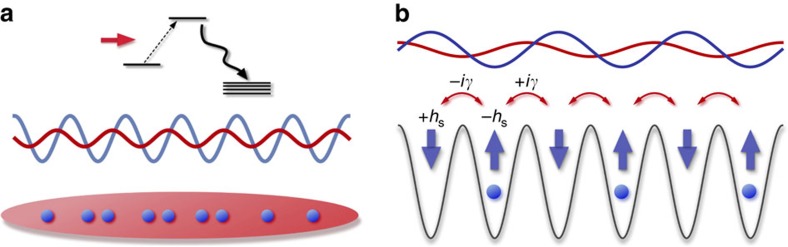
Experimental set-up of a PT-symmetric many-body system in ultracold atoms. (**a**) 1D ultracold atoms in a PT-symmetric optical lattice. Real (blue curve) and imaginary (red curve) parts of a complex potential are created by a pair of far-detuned and weak near-resonant standing waves. An imaginary potential results from a near-resonant light (red arrow) on atoms whose excited state has fast decay modes. The two periodic potentials are displaced from each other by one half of the lattice spacing so that the system possesses PT symmetry. (**b**) Mapping to a PT-symmetric lattice model that reproduces the same critical behaviour as the continuum model. Atoms are strongly localized by a deep optical lattice that does not affect the universal critical behaviour. The real and the imaginary parts of the complex potential introduce the on-site potentials ±*h*_s_ and imaginary hopping terms ±*iγ*. A lattice site occupied (not occupied) by a hard-core boson is represented by the up (down) spin.

The PT-symmetric many-body Hamiltonian 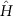 discussed above can be implemented in a 1D interacting ultracold bosonic atoms subject to a shallow PT-symmetric optical lattice *V*(*x*)=*V*_r_ cos(2*πx*/*d*)−*iV*_i_ sin(2*πx*/*d*), where *V*_r_ and *V*_i_ are the depths of the real and imaginary parts of a complex potential and *d* is the lattice constant. An imaginary optical potential can be realized by using a weak near-resonant standing-wave light (Methods section). Since *V*(*x*) remains invariant under simultaneous parity operation (*x*→−*x*) and time reversal (that is, complex conjugation), the system satisfies the condition of PT symmetry (Fig. 4a). In open quantum systems, by postselecting null measurement outcomes, the time evolution is governed by an effective non-Hermitian Hamiltonian^32^,^33^,^34^. The achieved experimental fidelity has already been high enough to allow experimenters to implement various types of postselections^35^,^36^,^37^. The low-energy behaviour of this system is then described by the PT-symmetric effective field theory 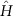. We note that the lattice Hamiltonian (4) can also be realized in ultracold atoms by superimposing a deep lattice that does not influence the universal critical behaviour (Fig. 4b).

We stress that the dynamics considered here is different from the one described by a master equation, where dissipative processes, in general, tend to destroy correlations underlying quantum critical phenomena. In contrast, the postselections allow us to study the system free from the dissipative jump processes, while non-trivial effects due to measurement backaction still occur via the non-Hermitian contributions in the effective Hamiltonian.

## Discussion

The reported fixed points in the extended parameter space suggest that an interplay between spectral singularity and quantum criticality results in an exotic universality class beyond the conventional paradigm. It remains an open question how the universality accompanying spectral singularity found in this work is related to non-unitary conformal field theories (CFT) studied in various fields ranging from statistical mechanics^38^ to high-energy physics^39^. It is particularly notable that a certain critical point of the integrable spin chain with PT-symmetric boundary fields corresponds to an exceptional point and is believed to be described by non-unitary CFT^40^. This suggests an intimate connection between the spectral singular critical point and the non-unitary CFT. Given recent success in measuring entanglement entropy in ultracold atoms^37^, it is of interest to study how quantum entanglement behaves in the presence of spectral singularity. In the PT-broken phase, we have shown that the ground state exhibits the enhanced superfluid correlation indicating the tighter binding of the topological excitations, in stark contrast to their proliferation as found in the BKT paradigm. In Hermitian systems, a relevant perturbation around RG fixed points has a tendency to suppress fluctuations of the concerned field and stabilize a non-critical, gapped phase. Our finding indicates that a relevant imaginary perturbation can realize the opposite situation of enhancing fluctuations of the concerned field and facilitating correlation in the conjugate field. An exploration of such unconventional quantum criticality in other synthetic, non-conservative many-body systems presents an interesting challenge. Further studies in these directions, together with their possible experimental realizations, could widen applications to future quantum metamaterials.

## Methods

### Details of numerical calculations

The phase diagram in Fig. 2a is determined from the exact diagonalization analysis of the lattice Hamiltonian (4). To identify the BKT transition point, we calculate the exact finite-size spectrum and find a crossing of low-energy levels having appropriate quantum numbers^28^. The PT transition point is identified as the first coalescence point in the low-energy spectrum with increasing *γ*. The calculations are done for different system sizes and the final results are obtained through extrapolation of the data to the thermodynamic limit. Further details are given in Supplementary Methods and Supplementary Fig. 4. The correlation function and the associated variation of the TLL parameter *K* shown in Fig. 3 are calculated by applying the iTEBD algorithm^31^. We emphasize that this method can be applied to study the ground-state properties of the non-Hermitian system. The method can accurately calculate the imaginary-time evolution 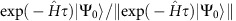 for an infinite system size, where *τ* is an imaginary time, 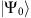 is an initial state and 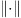 denotes the norm of the state. In the limit of large *τ*, we obtain the quantum state, the real part of which eigenvalue is the lowest in the entire spectrum, that is, an effective ground state of a non-Hermitian system. We note that the imaginary part of the eigenvalue does not affect the calculation since it only changes an overall phase of the wavefunction in the imaginary-time evolution. We then determine the TLL parameter *K* from the calculated correlation function by using the relation 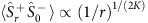.

### Derivation of the low-energy field theory of ultracold atoms

Here we explain the derivation of the low-energy effective field theory (1) of ultracold atoms. We start from the Hamiltonian in which the periodic potential *V*_r_ cos(2*πx*/*d*) is added to the Lieb–Liniger model^41^. Then, we introduce an imaginary optical lattice potential by using a weak near-resonant standing-wave light. This scheme is possible if the excited state 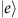 of an atom has decay modes other than the initial ground state 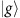 and its decay rate is faster than the spontaneous decay rate from 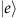 to 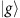 and the Rabi frequency^42^,^43^,^44^ (Fig. 4a). Such a condition can be satisfied by, for example, using appropriate atomic levels^45^ or light-induced transitions^16^. The difference between the wavelengths of the real and imaginary periodic potentials caused by different detunings of the lasers can be negligible. Using a second-order perturbation theory^8^ for the Rabi coupling and adiabatically eliminating the excited state, we obtain an effective time-evolution equation for the ground-state atoms. We then assume that null measurement outcomes are postselected so that the dynamics is described by the non-Hermitian Hamiltonian^32^,^33^,^34^. In this situation, the overall imaginary constant in the eigenvalue spectrum does not affect the dynamics since it can be eliminated when we normalize the quantum state, leading to the imaginary potential *iV*_i_ sin(2*πx*/*d*). Finally, we follow the standard procedure^22^ of taking the low-energy limit of the model and arrive at the Hamiltonian (1). The details of the calculations and experimental accessibility in ultracold atoms are described in Supplementary Notes 1 and 2.

### Data availability

The data that support the plots within this paper and other findings of this study are available from the corresponding author on request.

## Additional information

**How to cite this article:** Ashida, Y. *et al*. Parity-time-symmetric quantum critical phenomena. *Nat. Commun.* **8,** 15791 doi: 10.1038/ncomms15791 (2017).

**Publisher's note**: Springer Nature remains neutral with regard to jurisdictional claims in published maps and institutional affiliations.

## Supplementary Material

Supplementary Information Supplementary Notes, Supplementary Figures, Supplementary Methods, and Supplementary References
