# Supplementary material for: Parity-time-symmetric quantum critical phenomena
Source: Nat Commun. 2017 Jun 8;8:15791. doi: 10.1038/ncomms15791 (PMC5472709; doi:10.1038/ncomms15791)
Supplement: Supplementary Information — Supplementary Notes, Supplementary Figures, Supplementary Methods, and Supplementary References [file ncomms15791-s1.pdf]

Supplementary Note 1 - Parity-time symmetric effective Hamiltonian in ultracold atoms

Here we describe in detail how a parity-time (PT) symmetric field theory described by equation (1) in the main text can be implemented by using ultracold atoms.

We consider a situation in which atoms in the system have an energy level diagram shown in Supplementary Fig. 1. Here the excited state $|e\rangle$ has the frequency ω_0 relative to the ground state $|g\rangle$ and fast decay channels to other states with the total decay rate Γ much larger than the spontaneous emission rate from $|e\rangle$ to $|g\rangle$. The system is subject to a weak near-resonant light whose electric filed is given by $\mathbf{E}(\mathbf{x},t) = 2\mathbf{E}_0(\mathbf{x})\cos(\omega_{\rm L}t)$. The dynamics of atoms in the levels $\{|g\rangle, |e\rangle\}$ is then described by the many-body Lindblad equation:

$$\frac{\mathrm{d}\hat{\rho}}{\mathrm{d}t} = -\frac{i}{\hbar}[\hat{H},\hat{\rho}] - \frac{\Gamma}{2}\int\left[\hat{\Psi}_{\rm e}^{\dagger}(\mathbf{x})\hat{\Psi}_{\rm e}(\mathbf{x})\hat{\rho} + \hat{\rho}\hat{\Psi}_{\rm e}^{\dagger}(\mathbf{x})\hat{\Psi}_{\rm e}(\mathbf{x}) - 2\hat{\Psi}_{\rm e}(\mathbf{x})\hat{\rho}\hat{\Psi}_{\rm e}^{\dagger}(\mathbf{x})\right] d\mathbf{x}, \tag{1}$$

where $\hat{\Psi}_{\rm e}$ denotes the field operator of an excited atom and the terms involving Γ describe a loss of atoms in the state $|e\rangle$. Here $\hat{H}$ is the Hamiltonian of the interacting two-level atoms:

$$\hat{H} = \hat{\mathcal{H}}_{\rm g} + \hat{\mathcal{H}}_{\rm e} + \hat{\mathcal{V}}. \tag{2}$$

Going onto the rotating frame and making the rotating-wave approximation, the Hamiltonians $\hat{\mathcal{H}}_{\rm g}$ and $\hat{\mathcal{H}}_{\rm e}$ of ground- and excited-state atoms and the interaction Hamiltonian $\hat{\mathcal{V}}$

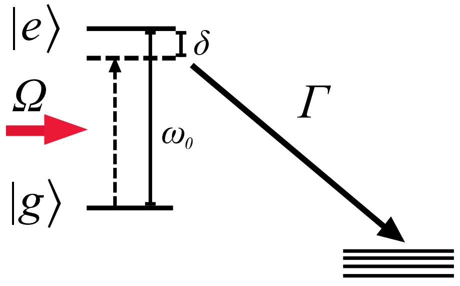

Supplementary Figure 1. **Energy-level diagram of an atom.** The excited state $|e\rangle$ has the frequency ω_0 relative to the ground state $|g\rangle$ and fast decay modes with the total decay rate Γ. A weak near-resonant light with the Rabi frequency Ω and detuning δ creates an effective imaginary potential for the ground-state atom, provided that Γ is much larger than the spontaneous decay rate from $|e\rangle$ to $|g\rangle$.

describing the Rabi coupling between the two atomic levels are given by

$$\hat{\mathcal{H}}_{\rm g} = \int d\mathbf{x} \left[\hat{\Psi}_{\rm g}^\dagger(\mathbf{x}) \left(-\frac{\hbar^2\nabla^2}{2m} + U_{\rm g}(\mathbf{x}) \right) \hat{\Psi}_{\rm g}(\mathbf{x}) + \frac{g}{2} \hat{\Psi}_{\rm g}^\dagger(\mathbf{x}) \hat{\Psi}_{\rm g}^\dagger(\mathbf{x}) \hat{\Psi}_{\rm g}(\mathbf{x}) \hat{\Psi}_{\rm g}(\mathbf{x}) \right], \tag{3}$$

$$\hat{\mathcal{H}}_{\rm e} = \int d\mathbf{x}\ \hat{\Psi}_{\rm e}^\dagger(\mathbf{x}) \left(-\frac{\hbar^2\nabla^2}{2m} + U_{\rm e}(\mathbf{x}) + \hbar\delta \right) \hat{\Psi}_{\rm e}(\mathbf{x}), \tag{4}$$

$$\hat{\mathcal{V}} = -\frac{\hbar}{2} \int d\mathbf{x} \left(\Omega(\mathbf{x}) \hat{\Psi}_{\rm g}^\dagger(\mathbf{x}) \hat{\Psi}_{\rm e}(\mathbf{x}) + \text{H.c.} \right) \equiv \hat{\mathcal{V}}_- + \hat{\mathcal{V}}_+, \tag{5}$$

where $U_{\rm g,e}(\mathbf{x})$'s are optical trapping potentials of the ground- and excited-state atoms created by a far-detuned light, g is the strength of the contact interaction between the ground-state atoms, $\delta = \omega_{\rm L} - \omega_0$ is the detuning, $\Omega(\mathbf{x}) = 2\mathbf{d} \cdot \mathbf{E}_0(\mathbf{x})/\hbar$ is the Rabi frequency with $\mathbf{d} = \langle e|\hat{\mathbf{d}}|g\rangle$ being the dipole moment, and $\hat{\mathcal{V}}_{+(-)}$ are the coupling terms that cause excitation (deexcitation) of the atoms. Let us introduce the non-Hermitian Hamiltonian $\hat{\mathcal{H}}_{\rm e,eff}$ of the excited-state atoms by

$$\hat{\mathcal{H}}_{\rm e,eff} = \hat{\mathcal{H}}_{\rm e} - \frac{i\hbar\Gamma}{2} \int d\mathbf{x} \hat{\Psi}_{\rm e}^\dagger(\mathbf{x}) \hat{\Psi}_{\rm e}(\mathbf{x}). \tag{6}$$

Then, the time-evolution equation (1) is written as follows:

$$\frac{{\rm d}\hat{\rho}}{{\rm d}t} = -\frac{i}{\hbar} \left[\left(\hat{\mathcal{H}}_{\rm g} + \hat{\mathcal{H}}_{\rm e,eff} + \hat{\mathcal{V}} \right) \hat{\rho} - \hat{\rho} \left(\hat{\mathcal{H}}_{\rm g} + \hat{\mathcal{H}}_{\rm e,eff}^\dagger + \hat{\mathcal{V}} \right) \right] + \Gamma \int d\mathbf{x} \hat{\Psi}_{\rm e}(\mathbf{x}) \hat{\rho} \hat{\Psi}_{\rm e}^\dagger(\mathbf{x}). \tag{7}$$

In the limit of rapid decay $\Gamma \gg \delta, \Omega$, we can adiabatically eliminate the rapidly evolving excited states and obtain the effective dynamics of the ground-state atoms. We achieve this by solving Supplementary Equation (7) using the second-order perturbation theory with respect to weak coupling $\hat{\mathcal{V}}$ [1]. As shown below, the resulting time-evolution equation for the ground-state atoms is given by Supplementary Equation (28), and it reduces to the effective non-Hermitian dynamics (33) with the effective Hamiltonian (29) when the postselection is implemented.

To perform the perturbative analysis, we work in the interaction picture, where the density matrix is given by

$$\hat{\tilde{\rho}}_{\rm I}(t) = e^{i\left(\hat{\mathcal{H}}_{\rm g} + \hat{\mathcal{H}}_{\rm e,eff}\right)t/\hbar} \hat{\rho}(t) e^{-i\left(\hat{\mathcal{H}}_{\rm g} + \hat{\mathcal{H}}_{\rm e,eff}^\dagger\right)t/\hbar}, \tag{8}$$

and a general operator $\hat{\mathcal{O}}$ is represented by

$$\hat{\mathcal{O}}_{\rm I}(t) = e^{i\left(\hat{\mathcal{H}}_{\rm g} + \hat{\mathcal{H}}_{\rm e,eff}\right)t/\hbar} \hat{\mathcal{O}} e^{-i\left(\hat{\mathcal{H}}_{\rm g} + \hat{\mathcal{H}}_{\rm e,eff}\right)t/\hbar}. \tag{9}$$

We note that $\hat{\tilde{\rho}}_{\rm I}$ in Supplementary Equation (8) is not normalized to unity in general. The time-evolution equation (7) is then simplified to

$$\dot{\hat{\tilde{\rho}}}_{\rm I} = -\frac{i}{\hbar} \left[\hat{\mathcal{V}}_{\rm I} \hat{\tilde{\rho}}_{\rm I} - \hat{\tilde{\rho}}_{\rm I} \hat{\mathcal{V}}_{\rm I}^\dagger \right] + \Gamma \int d\mathbf{x} \hat{\Psi}_{\rm I,e}(\mathbf{x}) \hat{\tilde{\rho}}_{\rm I} \hat{\Psi}_{\rm I,e}^\dagger(\mathbf{x}). \tag{10}$$

We assume that all the atoms reside in the ground state at $t = 0$. Then, we decompose the evolving state $\hat{\hat{\rho}}_{\rm I}(t)$ into a perturbation series with respect to the weak coupling $\hat{\mathcal{V}}_{\rm I}$:

$$\hat{\hat{\rho}}_{\rm I}(t) = \hat{\hat{\rho}}_{\rm I}^{(0)}(t) + \hat{\hat{\rho}}_{\rm I}^{(1)}(t) + \hat{\hat{\rho}}_{\rm I}^{(2)}(t) + \cdots, \qquad \left|\hat{\hat{\rho}}_{\rm I}^{(n)}(t)\right| \propto \left(\frac{|\Omega|}{\Gamma}\right)^n \left|\hat{\hat{\rho}}_{\rm I}^{(0)}(t)\right|, \tag{11}$$

where $|\cdots|$ denotes the trace norm. The recursive equations of the first three terms in the expansion (11) are given by

$$\dot{\hat{\hat{\rho}}}_{\rm I}^{(0)} = 0, \tag{12}$$

$$\dot{\hat{\hat{\rho}}}_{\rm I}^{(1)} = -\frac{i}{\hbar}\left[\hat{\mathcal{V}}_{\rm I}\hat{\hat{\rho}}_{\rm I}^{(0)} - \hat{\hat{\rho}}_{\rm I}^{(0)}\hat{\mathcal{V}}_{\rm I}^{\dagger}\right], \tag{13}$$

$$\dot{\hat{\hat{\rho}}}_{\rm I}^{(2)} = -\frac{i}{\hbar}\left[\hat{\mathcal{V}}_{\rm I}\hat{\hat{\rho}}_{\rm I}^{(1)} - \hat{\hat{\rho}}_{\rm I}^{(1)}\hat{\mathcal{V}}_{\rm I}^{\dagger}\right] + \Gamma\int d\mathbf{x}\hat{\Psi}_{\rm I,e}(\mathbf{x})\hat{\hat{\rho}}_{\rm I}^{(2)}\hat{\Psi}_{\rm I,e}^{\dagger}(\mathbf{x}). \tag{14}$$

From Supplementary Equation (12), we can take $\hat{\hat{\rho}}_{\rm I}^{(0)}$ as a time-independent operator. Supplementary Equation (13) can formally be integrated to give

$$\hat{\hat{\rho}}_{\rm I}^{(1)}(t) = -\frac{i}{\hbar}\int_0^t dt'\left[\hat{\mathcal{V}}_{\rm I}(t')\hat{\hat{\rho}}_{\rm I}^{(0)} - \hat{\hat{\rho}}_{\rm I}^{(0)}\hat{\mathcal{V}}_{\rm I}^{\dagger}(t')\right]. \tag{15}$$

To integrate out the excited states and obtain the effective dynamics of the ground-state atoms, we decompose $\hat{\hat{\rho}}_{\rm I}^{(2)}$ into the subspaces of the ground- and excited-state atoms. To do so, we introduce the projection $\hat{\mathcal{P}}_{\rm g}$ onto the ground-state manifold by $\hat{\mathcal{P}}_{\rm g} = \sum_N \hat{\mathcal{P}}_{\rm g}^N$, where $\hat{\mathcal{P}}_{\rm g}^N$ denotes the projection onto the subspace spanned by quantum states containing N ground-state atoms only. We also introduce the projection $\hat{\mathcal{Q}}_{\rm e}^1$ onto quantum states having a single excited-state atom (and an arbitrary number of ground-state atoms). Then, Supplementary Equation (14) can be decomposed as

$$\hat{\mathcal{P}}_{\rm g}\dot{\hat{\hat{\rho}}}_{\rm I}^{(2)}\hat{\mathcal{P}}_{\rm g} = -\frac{i}{\hbar}\hat{\mathcal{P}}_{\rm g}\left[\hat{\mathcal{V}}_{\rm I}\hat{\hat{\rho}}_{\rm I}^{(1)} - \hat{\hat{\rho}}_{\rm I}^{(1)}\hat{\mathcal{V}}_{\rm I}^{\dagger}\right]\hat{\mathcal{P}}_{\rm g} + \Gamma\hat{\mathcal{P}}_{\rm g}\int d\mathbf{x}\hat{\Psi}_{\rm I,e}\hat{\mathcal{Q}}_{\rm e}^1\hat{\hat{\rho}}_{\rm I}^{(2)}\hat{\mathcal{Q}}_{\rm e}^1\hat{\Psi}_{\rm I,e}^{\dagger}\hat{\mathcal{P}}_{\rm g}, \tag{16}$$

$$\hat{\mathcal{Q}}_{\rm e}^1\dot{\hat{\hat{\rho}}}_{\rm I}^{(2)}\hat{\mathcal{Q}}_{\rm e}^1 = -\frac{i}{\hbar}\hat{\mathcal{Q}}_{\rm e}^1\left[\hat{\mathcal{V}}_{\rm I}\hat{\hat{\rho}}_{\rm I}^{(1)} - \hat{\hat{\rho}}_{\rm I}^{(1)}\hat{\mathcal{V}}_{\rm I}^{\dagger}\right]\hat{\mathcal{Q}}_{\rm e}^1, \tag{17}$$

where Supplementary Equation (17) follows from the fact that $\hat{\hat{\rho}}_{\rm I}^{(2)}$ contains, at most, one excited-state atom. We adiabatically eliminate the excited states by integrating out Supplementary Equation (17):

$$\hat{\mathcal{Q}}_{\rm e}^1\hat{\hat{\rho}}_{\rm I}^{(2)}(t)\hat{\mathcal{Q}}_{\rm e}^1 = -\frac{i}{\hbar}\hat{\mathcal{Q}}_{\rm e}^1\int_0^t dt'\left[\hat{\mathcal{V}}_{\rm I}(t')\hat{\hat{\rho}}_{\rm I}^{(1)}(t') - \hat{\hat{\rho}}_{\rm I}^{(1)}(t')\hat{\mathcal{V}}_{\rm I}^{\dagger}(t')\right]\hat{\mathcal{Q}}_{\rm e}^1. \tag{18}$$

Substituting Supplementary Equations (15) and (18) into (16), we obtain

$$\begin{aligned}\hat{\mathcal{P}}_{\rm g}\dot{\hat{\hat{\rho}}}_{\rm I}^{(2)}\hat{\mathcal{P}}_{\rm g} = &-\frac{1}{\hbar^2}\hat{\mathcal{P}}_{\rm g}\left[\hat{\mathcal{V}}_{\rm I}(t)\int_0^t dt'\hat{\mathcal{V}}_{\rm I}(t')\hat{\hat{\rho}}_{\rm I}^{(0)} + \text{H.c.}\right]\hat{\mathcal{P}}_{\rm g} \\ &+\frac{\Gamma}{\hbar^2}\hat{\mathcal{P}}_{\rm g}\int d\mathbf{x}\hat{\Psi}_{\rm I,e}\hat{\mathcal{Q}}_{\rm e}^1\int_0^t dt'\int_0^{t'} dt''\left[\hat{\mathcal{V}}_{\rm I}(t')\hat{\hat{\rho}}_{\rm I}^{(0)}\hat{\mathcal{V}}_{\rm I}^{\dagger}(t'') + \text{H.c.}\right]\hat{\mathcal{Q}}_{\rm e}^1\hat{\Psi}_{\rm I,e}^{\dagger}\hat{\mathcal{P}}_{\rm g}.\end{aligned} \tag{19}$$

Here, in the second line in Supplementary Equation (19), the terms proportional to $\hat{\mathcal{V}}_{\rm I}\hat{\mathcal{V}}_{\rm I}\hat{\tilde{\rho}}_{\rm I}^{(0)}$ or $\hat{\tilde{\rho}}_{\rm I}^{(0)}\hat{\mathcal{V}}_{\rm I}^{\dagger}\hat{\mathcal{V}}_{\rm I}^{\dagger}$ vanish because of the projection $\hat{\mathcal{Q}}_{\rm e}^{1}$. Then, since we assume that the time scale of the strong dissipation is fast compared with other time scales appearing in the system, we approximate the leading contributions by

$$e^{-i\left(\hat{\mathcal{H}}_{\rm g}+\hat{\mathcal{H}}_{\rm e,eff}\right)t/\hbar}\hat{\mathcal{P}}_{\rm g}\simeq\hat{\mathcal{P}}_{\rm g},\quad e^{-i\left(\hat{\mathcal{H}}_{\rm g}+\hat{\mathcal{H}}_{\rm e,eff}\right)t/\hbar}\hat{\mathcal{Q}}_{\rm e}^{1}\simeq e^{-\Gamma t/2}\hat{\mathcal{Q}}_{\rm e}^{1}. \tag{20}$$

From these equations, it follows that

$$\begin{aligned}\hat{\mathcal{P}}_{\rm g}\hat{\mathcal{V}}_{\rm I}(t)&=\hat{\mathcal{P}}_{\rm g}e^{i\left(\hat{\mathcal{H}}_{\rm g}+\hat{\mathcal{H}}_{\rm e,eff}\right)t/\hbar}(\hat{\mathcal{V}}_{+}+\hat{\mathcal{V}}_{-})e^{-i\left(\hat{\mathcal{H}}_{\rm g}+\hat{\mathcal{H}}_{\rm e,eff}\right)t/\hbar}\\&\simeq\hat{\mathcal{P}}_{\rm g}\hat{\mathcal{V}}_{-}\hat{\mathcal{Q}}_{\rm e}^{1}e^{-i\left(\hat{\mathcal{H}}_{\rm g}+\hat{\mathcal{H}}_{\rm e,eff}\right)t/\hbar}\\&\simeq e^{-\Gamma t/2}\hat{\mathcal{P}}_{\rm g}\hat{\mathcal{V}}_{-}\hat{\mathcal{Q}}_{\rm e}^{1}.\end{aligned} \tag{21}$$

Similarly, we obtain

$$\begin{aligned}\hat{\mathcal{Q}}_{\rm e}^{1}\hat{\mathcal{V}}_{\rm I}(t)\hat{\mathcal{P}}_{\rm g}&=\hat{\mathcal{Q}}_{\rm e}^{1}e^{i\left(\hat{\mathcal{H}}_{\rm g}+\hat{\mathcal{H}}_{\rm e,eff}\right)t/\hbar}(\hat{\mathcal{V}}_{+}+\hat{\mathcal{V}}_{-})e^{-i\left(\hat{\mathcal{H}}_{\rm g}+\hat{\mathcal{H}}_{\rm e,eff}\right)t/\hbar}\hat{\mathcal{P}}_{\rm g}\\&\simeq e^{\Gamma t/2}\hat{\mathcal{Q}}_{\rm e}^{1}\hat{\mathcal{V}}_{+}\hat{\mathcal{P}}_{\rm g}\end{aligned} \tag{22}$$

We then perform the integration in the first line on the right-hand side of Supplementary Equation (19) and obtain

$$\begin{aligned}-\frac{1}{\hbar^2}\hat{\mathcal{P}}_{\rm g}\left[\hat{\mathcal{V}}_{\rm I}(t)\int_0^t dt'\hat{\mathcal{V}}_{\rm I}(t')\hat{\tilde{\rho}}_{\rm I}^{(0)}+{\rm H.c.}\right]\hat{\mathcal{P}}_{\rm g}&\simeq-\frac{1}{\hbar^2}\left[\hat{\mathcal{P}}_{\rm g}\hat{\mathcal{V}}_{-}\hat{\mathcal{Q}}_{\rm e}^{1}e^{-\Gamma t/2}\int_0^t dt' e^{\Gamma t'/2}\hat{\mathcal{Q}}_{\rm e}^{1}\hat{\mathcal{V}}_{+}\hat{\mathcal{P}}_{\rm g}\hat{\tilde{\rho}}_{\rm I}^{(0)}+{\rm H.c.}\right]\\&\simeq-\frac{2}{\hbar^2\Gamma}\left(\hat{\mathcal{P}}_{\rm g}\hat{\mathcal{V}}_{-}\hat{\mathcal{V}}_{+}\hat{\mathcal{P}}_{\rm g}\hat{\tilde{\rho}}_{\rm I}^{(0)}+\hat{\tilde{\rho}}_{\rm I}^{(0)}\hat{\mathcal{P}}_{\rm g}\hat{\mathcal{V}}_{-}\hat{\mathcal{V}}_{+}\hat{\mathcal{P}}_{\rm g}\right)\\&=-\left\{\int d\mathbf{x}\frac{|\Omega(\mathbf{x})|^2}{2\Gamma}\hat{\Psi}_{\rm g}^{\dagger}(\mathbf{x})\hat{\Psi}_{\rm g}(\mathbf{x}),\hat{\tilde{\rho}}_{\rm I}^{(0)}\right\},\end{aligned} \tag{23}$$

where we use Supplementary Equations (21), (22), and the relations $\hat{\mathcal{P}}_{\rm g}\hat{\tilde{\rho}}_{\rm I}^{(0)}\hat{\mathcal{P}}_{\rm g}=\hat{\tilde{\rho}}_{\rm I}^{(0)}$ and $(\hat{\mathcal{Q}}_{\rm e}^{1})^2=\hat{\mathcal{Q}}_{\rm e}^{1}$ in the first line, and use Supplementary Equation (5) to derive the last line. To calculate the last line in Supplementary Equation (19), we approximate

$$\begin{aligned}\hat{\mathcal{Q}}_{\rm e}^{1}\int_0^t dt'\int_0^{t'}dt''\left[\hat{\mathcal{V}}_{\rm I}(t')\hat{\tilde{\rho}}_{\rm I}^{(0)}\hat{\mathcal{V}}_{\rm I}^{\dagger}(t'')+{\rm H.c.}\right]\hat{\mathcal{Q}}_{\rm e}^{1}&\simeq2\int_0^t dt'\int_0^{t'}dt'' e^{\Gamma(t'+t'')/2}\hat{\mathcal{Q}}_{\rm e}^{1}\hat{\mathcal{V}}_{+}\hat{\tilde{\rho}}_{\rm I}^{(0)}\hat{\mathcal{V}}_{-}\hat{\mathcal{Q}}_{\rm e}^{1}\\&\simeq\frac{4e^{\Gamma t}}{\Gamma^2}\hat{\mathcal{V}}_{+}\hat{\tilde{\rho}}_{\rm I}^{(0)}\hat{\mathcal{V}}_{-},\end{aligned} \tag{24}$$

and

$$\hat{\mathcal{P}}_{\rm g}\hat{\Psi}_{\rm I,e}\hat{\mathcal{Q}}_{\rm e}^{1}\simeq e^{-\Gamma t/2}\hat{\mathcal{P}}_{\rm g}\hat{\Psi}_{\rm e}\hat{\mathcal{Q}}_{\rm e}^{1},\quad\hat{\mathcal{Q}}_{\rm e}^{1}\hat{\Psi}_{\rm I,e}^{\dagger}\hat{\mathcal{P}}_{\rm g}\simeq e^{-\Gamma t/2}\hat{\mathcal{Q}}_{\rm e}^{1}\hat{\Psi}_{\rm e}^{\dagger}\hat{\mathcal{P}}_{\rm g} \tag{25}$$

The last line in Supplementary Equation (19) can then be calculated as

$$\begin{aligned}
&\frac{\Gamma}{\hbar^2}\hat{\mathcal{P}}_{\rm g}\int d\mathbf{x}\hat{\Psi}_{\rm I,e}\hat{\mathcal{Q}}_{\rm e}^{1}\int_0^t dt'\int_0^{t'} dt''\left[\hat{\mathcal{V}}_{\rm I}(t')\hat{\tilde{\rho}}_{\rm I}^{(0)}\hat{\mathcal{V}}_{\rm I}^{\dagger}(t'')+{\rm H.c.}\right]\hat{\mathcal{Q}}_{\rm e}^{1}\hat{\Psi}_{\rm I,e}^{\dagger}\hat{\mathcal{P}}_{\rm g}\\
&\simeq\frac{4}{\hbar^2\Gamma}\int d\mathbf{x}\hat{\mathcal{P}}_{\rm g}\hat{\Psi}_{\rm e}(\mathbf{x})\hat{\mathcal{V}}_{+}\hat{\mathcal{P}}_{\rm g}\hat{\tilde{\rho}}_{\rm I}^{(0)}\hat{\mathcal{P}}_{\rm g}\hat{\mathcal{V}}_{-}\hat{\Psi}_{\rm e}^{\dagger}(\mathbf{x})\hat{\mathcal{P}}_{\rm g}\\
&\simeq\hat{\mathcal{P}}_{\rm g}\int d\mathbf{x}\frac{|\Omega(\mathbf{x})|^2}{\Gamma}\hat{\Psi}_{\rm g}(\mathbf{x})\hat{\tilde{\rho}}_{\rm I}^{(0)}\hat{\Psi}_{\rm g}^{\dagger}(\mathbf{x})\hat{\mathcal{P}}_{\rm g},
\end{aligned}\tag{26}$$

where we use Supplementary Equations (24) and (25) and $\hat{\mathcal{P}}_{\rm g}\hat{\tilde{\rho}}_{\rm I}^{(0)}\hat{\mathcal{P}}_{\rm g}=\hat{\tilde{\rho}}_{\rm I}^{(0)}$ in the second line. To derive the last line, we use the following relation

$$\hat{\mathcal{P}}_{\rm g}\hat{\Psi}_{\rm e}(\mathbf{x})\hat{\mathcal{V}}_{+}\hat{\mathcal{P}}_{\rm g}=-\frac{\hbar\Omega^*(\mathbf{x})}{2}\hat{\mathcal{P}}_{\rm g}\hat{\Psi}_{\rm g}(\mathbf{x})\hat{\mathcal{P}}_{\rm g}.\tag{27}$$

From equations (19), (23), (26) and $\hat{\mathcal{P}}_{\rm g}\dot{\hat{\tilde{\rho}}}_{\rm I}^{(0)}\hat{\mathcal{P}}_{\rm g}=\hat{\mathcal{P}}_{\rm g}\dot{\hat{\tilde{\rho}}}_{\rm I}^{(1)}\hat{\mathcal{P}}_{\rm g}=0$, the effective time-evolution equation of the ground-state atoms is obtained as

$$\frac{{\rm d}\hat{\rho}_{\rm g}}{{\rm d}t}=-\frac{i}{\hbar}\left(\hat{\mathcal{H}}_{\rm g,eff}\hat{\rho}_{\rm g}-\hat{\rho}_{\rm g}\hat{\mathcal{H}}_{\rm g,eff}^{\dagger}\right)+\int d\mathbf{x}\frac{|\Omega(\mathbf{x})|^2}{\Gamma}\hat{\Psi}_{\rm g}(\mathbf{x})\hat{\rho}_{\rm g}\hat{\Psi}_{\rm g}^{\dagger}(\mathbf{x}),\tag{28}$$

$$\hat{\mathcal{H}}_{\rm g,eff}\equiv\hat{\mathcal{H}}_{\rm g}-i\hbar\int d\mathbf{x}\frac{|\Omega(\mathbf{x})|^2}{2\Gamma}\hat{\Psi}_{\rm g}^{\dagger}(\mathbf{x})\hat{\Psi}_{\rm g}(\mathbf{x}),\tag{29}$$

where we go back to the Schrödinger picture and introduce the density matrix $\hat{\rho}_{\rm g}$ projected onto the ground-state manifold by

$$\hat{\rho}_{\rm g}(t)=\hat{\mathcal{P}}_{\rm g}\hat{\rho}(t)\hat{\mathcal{P}}_{\rm g}\simeq\hat{\mathcal{P}}_{\rm g}\left(\hat{\rho}^{(0)}(t)+\hat{\rho}^{(1)}(t)+\hat{\rho}^{(2)}(t)\right)\hat{\mathcal{P}}_{\rm g}.\tag{30}$$

The non-Hermitian Hamiltonian (29) describes the effective dynamics of the system when we postselect realizations in which no quantum jumps occur, i.e., no atoms escape from the ground state [2–4]. To clarify this point, let us assume that N ground-state atoms are initially prepared, i.e., $\hat{\mathcal{P}}_{\rm g}^{N}\hat{\rho}(0)\hat{\mathcal{P}}_{\rm g}^{N}=\hat{\rho}(0)$. This initial condition implies

$$\hat{\mathcal{P}}_{\rm g}^{N+l}\hat{\rho}_{\rm g}(0)\hat{\mathcal{P}}_{\rm g}^{N+l}=0\qquad(l=1,2,\ldots),\tag{31}$$

where we use $\left[\hat{\mathcal{P}}_{\rm g}^{N},\hat{\mathcal{P}}_{\rm g}\right]=0$. From Supplementary Equations (28) and (31), we can in particular show that, during the course of the time evolution,

$$\hat{\mathcal{P}}_{\rm g}^{N+1}\hat{\rho}_{\rm g}(t)\hat{\mathcal{P}}_{\rm g}^{N+1}=0.\tag{32}$$

Let us now consider the dynamics of the postselected system $\hat{\tilde{\rho}}_{\rm g}^{N}(t)\equiv\hat{\mathcal{P}}_{\rm g}^{N}\hat{\rho}(t)\hat{\mathcal{P}}_{\rm g}^{N}=\hat{\mathcal{P}}_{\rm g}^{N}\hat{\rho}_{\rm g}(t)\hat{\mathcal{P}}_{\rm g}^{N}$, where the dynamics is conditioned such that no atoms are lost from the

initial state. Using Supplementary Equations (28) and (32), we can show that the dynamics of the postselected system $\hat{\tilde{\rho}}_{\mathrm{g}}^N$ is governed by the non-Hermitian Hamiltonian (29):

$$\frac{\mathrm{d}\hat{\tilde{\rho}}_{\mathrm{g}}^N}{\mathrm{d}t} = -\frac{i}{\hbar}\left(\hat{\mathcal{H}}_{\mathrm{g,eff}}\hat{\tilde{\rho}}_{\mathrm{g}}^N - \hat{\tilde{\rho}}_{\mathrm{g}}^N\hat{\mathcal{H}}_{\mathrm{g,eff}}^{\dagger}\right). \tag{33}$$

Some remarks are in order here. First, an imaginary potential $-i|\Omega(\mathbf{x})|^2/(2\Gamma)$ in Supplementary Equation (29) arises from the second-order process of a virtual excitation and de-excitation of the ground-state atoms (see Supplementary Equation (23)). Since no atoms are lost in this process, the non-Hermitian contribution exists even when we do not observe actual losses of atoms. Physically, such a contribution originates from the measurement backaction associated with continuous monitoring of the population of atoms in the excited state [2]. Second, we note that the expression of the imaginary potential indicates that the loss rate of atoms from the ground state is suppressed by a factor of Ω/Γ for large Γ. In particular, in the limit of $\Gamma \to \infty$, the dynamics reduces to the Hermitian evolution governed by $\hat{\mathcal{H}}_{\mathrm{g}}$. This limit can be interpreted as the quantum Zeno dynamics [5], where the strong measurement confines the dynamics into the decay-free subspace and the time-evolution obeys the effective "Zeno" Hamiltonian. In our model, such a Hamiltonian is given by $\hat{\mathcal{H}}_{\mathrm{g}} = \hat{\mathcal{P}}_{\mathrm{g}}\hat{H}\hat{\mathcal{P}}_{\mathrm{g}}$, where the total Hamiltonian $\hat{H}$ is projected onto the decay-free, ground-state manifold. In a general case of a strong but finite Γ, we need to perform careful perturbative analyses [6–9] to obtain the correction terms beyond the quantum Zeno dynamics, as we have conducted above.

We are now in a position to derive the PT-symmetric Hamiltonian. We consider a system confined in a one-dimensional (1D) optical trap, and assume that the system is subject to a real shallow periodic potential (optical lattice), $U(x) = V_{\mathrm{r}}\cos(2\pi x/d)$, which can be created by a far-detuned off-resonant light. Here V_{r} is the lattice potential which is controlled by changing the intensity of the light, and $d = \lambda/2$ is the lattice spacing. We then superimpose the near-resonant standing-wave light discussed above with the displacement of $d/4$. We thus have $\mathbf{E}_0(x) = \mathcal{E}_0\cos(kx - \pi/4)$ with $k = 2\pi/\lambda$. From Supplementary Equations (3) and (29), the resulting Hamiltonian is obtained as

$$\hat{\mathcal{H}}_{\mathrm{eff}} = \int dx \hat{\Psi}^{\dagger}(x)\left(-\frac{\hbar^2\nabla^2}{2m} + V(x)\right)\hat{\Psi}(x) + \frac{g}{2}\int dx \hat{\Psi}^{\dagger}(x)\hat{\Psi}^{\dagger}(x)\hat{\Psi}(x)\hat{\Psi}(x), \tag{34}$$

where we drop the subscript g and introduce

$$V(x) = V_{\rm r} \cos\left(\frac{2\pi x}{d}\right) - iV_{\rm i} \sin\left(\frac{2\pi x}{d}\right), \tag{35}$$

$$V_{\rm i} = \frac{|d|^2\mathcal{E}_0^2}{\hbar\Gamma}. \tag{36}$$

Here we redefine the interaction parameter g by incorporating the renormalization factor due to the 1D confinement and ignore the constant term $-iV_{\rm i}N$ proportional to the total number of atoms. This constant term is irrelevant in the postselected dynamics here because it is cancelled upon the normalization of the quantum state. The effective Hamiltonian (34) satisfies PT symmetry because the potential satisfies the condition $V(x) = V^*(-x)$. While we here assume that the wavelengths of the lasers creating the real and imaginary potentials in Supplementary Equation (35) are the same, this assumption can be well met because the detuning required for the real potential causes only a negligible shift in λ, as detailed later.

Finally, we derive the low-energy effective theory. An interacting 1D Bose gas as described by Supplementary Equation (34) without the potential $V(x)$ is described at low energies by the Tomonaga-Luttinger liquid theory [10]:

$$\hat{H}_{\rm TLL} = \int dx \frac{\hbar v}{2\pi}\left[K(\partial_x\hat{\theta})^2 + \frac{1}{K}(\partial_x\hat{\phi})^2\right]. \tag{37}$$

Here $\hat{\theta}$ is related to the phase of the bosonic field operator $\hat{\Psi}^\dagger(x) = \sqrt{\hat{\rho}(x)}e^{-i\hat{\theta}(x)}$, and $\hat{\phi}$ is related to the density operator as

$$\hat{\rho}(x) = \left[\rho_0 - \frac{1}{\pi}\partial_x\hat{\phi}\right]\sum_{p=-\infty}^{\infty} e^{2ip(\pi\rho_0 x - \hat{\phi}(x))}, \tag{38}$$

where ρ_0 is the average atomic density. We discuss the perturbative role of the potential term

$$\hat{H}_V = \int dx\ V(x)\hat{\Psi}^\dagger(x)\hat{\Psi}(x) = \int dx\ V(x)\hat{\rho}(x). \tag{39}$$

Since we are interested in the commensurate phase transition, we assume the unit filling $\rho_0 d = 1$, i.e., one atom per site. By substituting Supplementary Equations (35) and (38) into Supplementary Equation (39) and ignoring fast oscillating terms, we obtain [11]

$$\hat{H}_V = \rho_0 V_{\rm r}\int dx \cos\left[2\hat{\phi}(x)\right] - i\rho_0 V_{\rm i}\int dx \sin\left[2\hat{\phi}(x)\right]. \tag{40}$$

Defining $\alpha_{\rm r,i} \equiv \pi\rho_0 V_{\rm r,i}$, we arrive at the PT-symmetric potential term in equation (2) in the main text.

Supplementary Note 2 - Experimental implementation and signatures in ultracold atoms

We here describe detailed experimental signatures of the PT-symmetric system in ultracold atoms. To create an imaginary optical potential, we need to realize atomic levels as illustrated in Supplementary Fig. 1. The fast decay modes can be realized by choosing (i) appropriate spontaneous emission processes or (ii) light-induced transitions. In the scheme (i), one can use the $F = 3$ to $F' = 3$ transition ($5S_{1/2}$ to $5P_{3/2}$) of ^{85}Rb atoms to create an imaginary potential [12], where the excited $F' = 3$ state has a decay channel to the $F = 2$ state. Implementations of complex potentials have also been demonstrated by using other metastable atomic states [13–15]. The postselection of the null measurement outcomes can be implemented by, e.g., applying the state-selective imaging technique [16, 17]. Here we first load the ground-state atoms into a real optical potential with an accurately estimated number of atoms. Such a preparation has been achieved with the single-site addressing technique in ultracold atomic experiments [18, 19]. Then we ramp up an imaginary potential and let the system evolve in time. When an atom is excited by a weak near-resonant light, it quickly decays into modes other than the original ground state. Thus, the postselection can be realized by applying the state-selective imaging at the final stage of the time evolution, thereby measuring the number of atoms residing in the ground state, and selecting the realizations in which this number is unchanged between the initial and final states. In this way, we can postselect processes with null quantum jump. We note that the experimental fidelity of measuring the atom number with such site-resolved imaging has reached almost unit fidelity (99.5% according to Ref. [20]). In view of this development, we expect that the postselection process as described above can be performed with near-unit fidelity. We note that various types of postselections have already been achieved owing to the high experimental fidelity [17, 19, 21].

We next discuss the scheme (ii) that exploits light-induced transitions. Here, the level structures for creating an imaginary potential can be obtained by inducing a fluorescent transition between the excited state and a state other than the original ground state. In this setup, when the ground-state atom is excited, it is quickly lost from an optical potential because the recoil energy due to fluorescent imaging light causes heating of atoms. However, only a few tens of scattered photons are enough for the loss of those atoms [22] and thus

creating an imaginary potential. If the resulting loss rate Γ is much larger than both the spontaneous emission rate and the Rabi frequency Ω, we can adiabatically eliminate the excited state and implement an effective imaginary potential [13]. The postselection of the null outcome can be realized, for example, by continuously monitoring the system with quantum gas microscopy. If an atom is excited during the time evolution, it emits photons which can be detected by quantum gas microscopy [20, 23]. Thus, by selecting the events in which no atoms emit photons, the postselection of null quantum jumps (no atomic loss) can be realized. We may also double check the postselection process by performing a projective atom-number measurement at the final stage of the time evolution and ensuring that the atom number stays constant during the time evolution, as described above.

While substantial atomic losses usually lead to experimental difficulties, our theoretical predictions are accessible by using a very weak imaginary potential with which the atomic loss rate can be made arbitrarily small. This is because the key parameter which drives the phase transitions discussed in the main text is the ratio between g_{i} and g_{r}, which is equivalent to the ratio between the amplitudes of the imaginary and real potentials (see Supplementary Equation (40)), and the imaginary potential required to induce the transition can be made very weak if the depth of the real part of the optical potential is chosen to be sufficiently small. Indeed, such a weak imaginary potential can be implemented in our model since the atomic loss rate is suppressed by a factor of Ω/Γ in the limit of large Γ (see Supplementary Equation (29)).

Since the depth of the real potential can be made small, and the condition on the Rabi frequency $\Delta_{\mathrm{off}} > \Omega$ can easily be met owing to the smallness of the optical depth, the only requirement for the detuning Δ_{off} of the off-resonant light for the real part of the optical potential is $\Delta_{\mathrm{off}} > \Gamma$. This point validates our assumption that the real and imaginary potentials have the same periodicity. For example, for (i) the spontaneous emission process in ^{85}Rb or (ii) the light-induced transition in the $D2$ transition of ^{87}Rb, Γ is of the order of tens of MHz [12, 22]. Thus, if we set the detuning at $\Delta_{\mathrm{off}} = 100$ GHz, the off-resonant condition is well satisfied, while such a detuning causes a less than 0.1% shift in the optical wavelength.

We finally discuss experimental signatures of the theoretical predictions described in the main text. First, the measurement-induced Berezinskii-Kosterlitz-Thouless (BKT) transition corresponds to a 1D superfluid-to-Mott-insulator transition for ultracold atoms. This

is associated with a power-law divergence in the momentum distribution at zero momentum [11], which can be detected by applying the standard techniques such as time-of-flight imaging [24]. Second, the PT symmetry breaking can be probed by detecting the single-mode lasing dynamics of the system. In the PT-broken region, the system has an excited state whose eigenvalue possesses a positive imaginary contribution; such an excited state would have an exponentially growing amplitude in the time evolution. Thus, after exciting the system through, e.g., shaking of an optical lattice [25], the system eventually approaches the state having the largest imaginary part of the eigenvalue. Such a single-mode lasing dynamics entails a significant decrease in the entropy of the system, which can be probed from shot-to-shot fluctuations in *in-situ* imaging of atomic gases [17, 19, 21, 25]. Third, the anomalous variation of the critical exponent shown in Fig. 3 in the main text can be investigated through the analysis of the shot-to-shot noise correlations in density fluctuations of a 1D Bose gas, as demonstrated in Ref. [26].

Supplementary Note 3 - Preparation of the ground state in the PT-symmetric spin-chain model

Let us here discuss how we can study the ground state of the PT-symmetric spin-chain model in the PT-broken regime. When the PT symmetry is broken, some excited eigenstates turn out to have complex pairs of eigenvalues while the ground state remains to have a real eigenvalue (see Supplementary Fig. 2(a)-(d) for typical spectra). In particular, there exist high-lying unstable modes having positive imaginary parts of eigenvalues. As a result, if the system is significantly perturbed and highly excited, the amplitudes of these modes can grow in time and eventually govern the physical properties of the system. This is reminiscent of the phenomenon known as parametric instability or self-pulsing [27] in excition-polariton systems, which in general destroys the off-diagonal quasi-long-range order in 1D Bose systems [28–30].

In contrast, our main focus here is on the ground state that sustains the quantum critical behavior. This state is indeed relevant in our setup, where the system is initially prepared in the zero-temperature state of the hermitian Hamiltonian and then the imaginary part of the potential required for the PT symmetry is adiabatically ramped up. We numerically demonstrate in Supplementary Fig. 3 that the system remains in the ground state with almost unit fidelity for a long time interval. Here we consider the spin-chain model (equa-

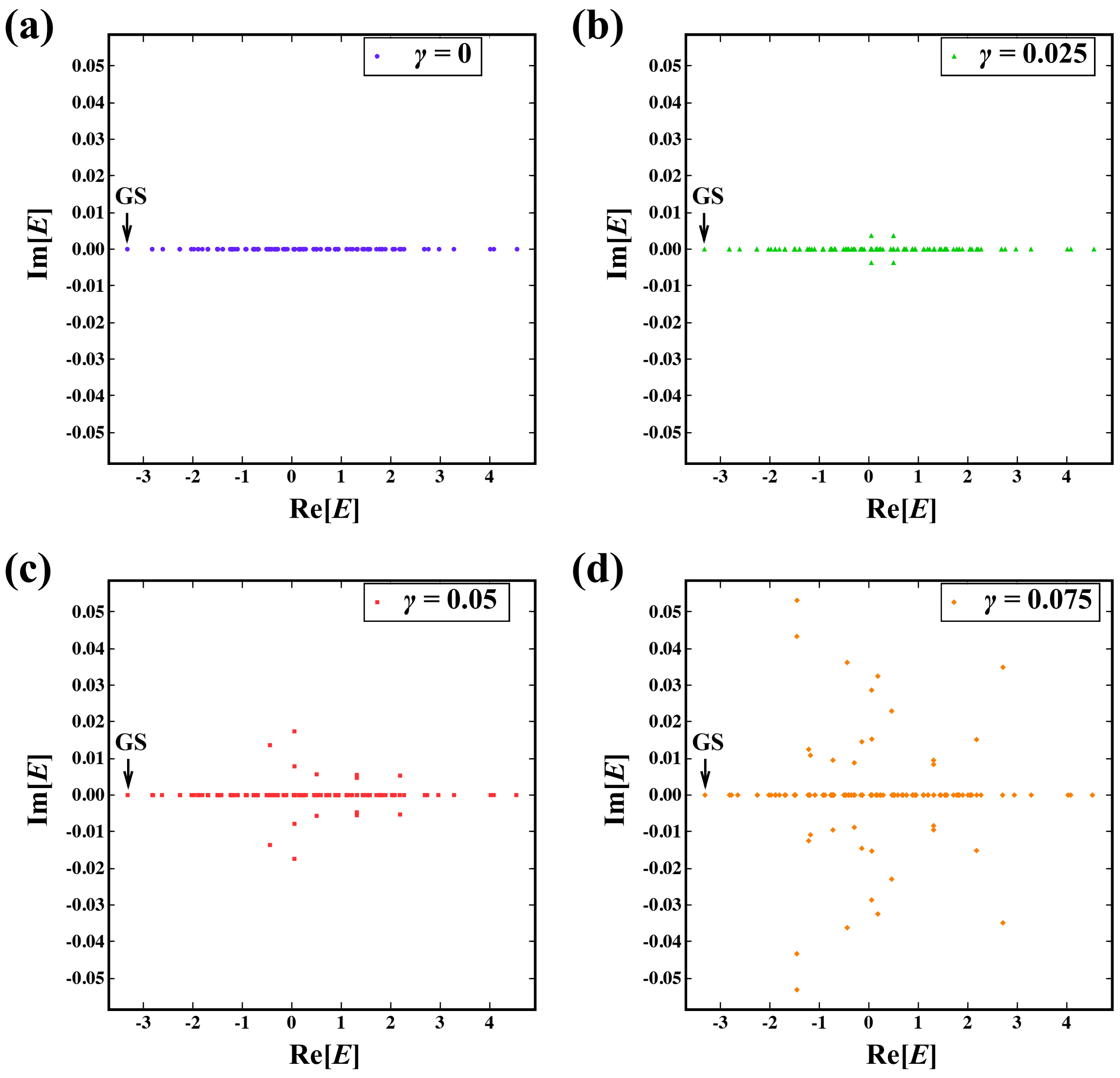

Supplementary Figure 2. **Exact finite-size spectra.** The spectra of the lattice spin-chain model are plotted with the parameters $-\Delta = 0.5, h_s = 0.0, N = 12$ for different strengths of the imaginary hopping (a) $\gamma = 0$, (b) $\gamma = 0.025$, (c) $\gamma = 0.05$, and (d) $\gamma = 0.075$. When the PT symmetry is broken, some of excited states have pairs of complex eigenvalues which are conjugate to each other, while the ground state remains to have a real eigenvalue. The plotted energy levels reside in the sector $(S^z = 0, q = 0, P = T = 1)$. The ground state (GS) is indicated by the black arrow.

tion (4) in the main text) and adiabatically ramp up the imaginary term with the time dependence $\gamma(t) = \gamma \times \left(1 - 2/\left(e^{(t/\tau)^2} + 1\right)\right)$, where τ characterizes the timescale of the operation. The initial state $|\Psi(0)\rangle$ is chosen to be the ground state of the Hamiltonian with $\gamma(0) = 0$, and the time evolution $|\Psi(t)\rangle$ is calculated by diagonalizing the Hamiltonian at

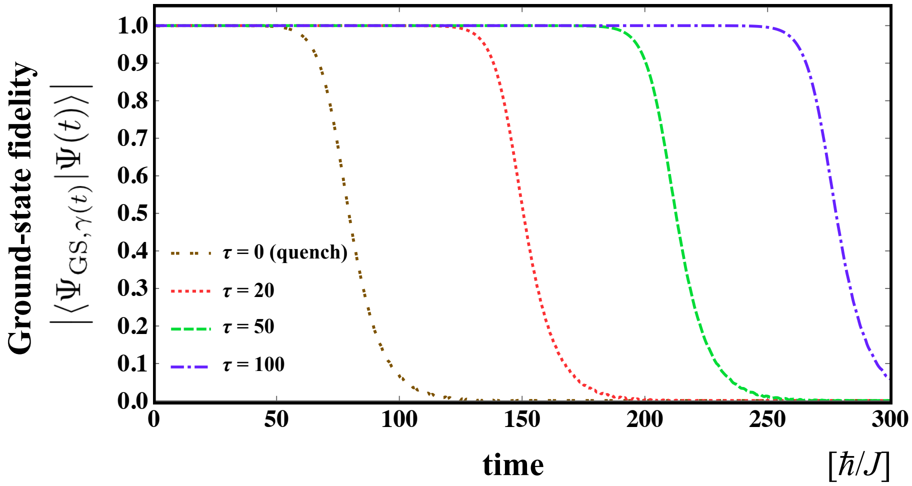

Supplementary Figure 3. **Ground-state fidelity in the PT-broken regime.** The time evolution of the ground-state fidelity of the system in the PT-broken regime is plotted for several different values of τ. The imaginary hopping term γ is ramped up with different timescales $\tau = 0, 20, 50, 100$. The ground state $|\Psi_{\mathrm{GS},\gamma(t)}\rangle$ is calculated from the exact diagonalization of the Hamiltonian at each time step. The parameters are set to $-\Delta = 0.5$, $h_s = 0$, $\gamma = 0.05$, and $N = 12$.

each time step. Supplementary Figure 3 shows the ground-state fidelity $|\langle\Psi_{\mathrm{GS},\gamma(t)}|\Psi(t)\rangle|$ of the instantaneous Hamiltonian with $\gamma(t)$, indicating that the system remains in the ground state for a time much longer than the ramping time τ. Using a typical experimental time scale $\hbar/J = 3.6/(2\pi)$ ms [31], the lifetime of the ground state can reach ~ 150 ms, which is sufficiently long compared with a typical operation time of ultracold atom experiments [31]. We note that the first signature of the enhancement of superfluid correlation can appear from a relatively small size such as ~ 10 sites (see Fig. 3b in the main text).

Supplementary Methods - Determination of the phase diagram of the lattice model

Here we describe in detail the methods for determining the BKT and PT phase boundaries in the lattice model (equation (4) in the main text). We first describe the so-called level spectroscopy method [32, 33], which has been developed to accurately determine the BKT transition point. The key idea of this method is to relate the low-energy spectrum to the running coupling constants that appear in the renormalization group (RG) equations. Under the periodic boundary condition, the lattice Hamiltonian is invariant with respect to spin rotation about the z axis, translation by two sites, space inversion, and spin reversal. The corresponding conserved quantum numbers are the total magnetization $S^z \equiv \sum_{i=1}^{N} S_{\mathrm{i}}^z$, the wavenumber $q = 2\pi k/L$ ($k \in \mathbb{Z}, L \equiv N/2$), the parity $P = \pm 1$, and the spin reversal $T = \pm 1$. The ground state with energy $E_{\mathrm{g}}(L)$ resides in the sector ($S^z = 0, q = 0, P = T = 1$). Following Ref. [33], we denote the second lowest eigenenergy in this sector by E_0 and the lowest eigenenergy in the sector ($S^z = \pm 4, q = 0, P = 1$) by E_3. Near the BKT transition line, these excitation energies satisfy [32]

$$E_0(L) - E_{\mathrm{g}}(L) = \frac{2\pi v}{L}\left(2 + \frac{1}{3}\delta(l) - \frac{8}{3}g'(l)\right), \tag{41}$$

$$E_3(L) - E_{\mathrm{g}}(L) = \frac{2\pi v}{L}(2 - \delta(l)), \tag{42}$$

where $\delta \equiv K - 2$, $g' \equiv \sqrt{g_{\mathrm{r}}^2 - g_{\mathrm{i}}^2}$, and the logarithmic RG scale l is related to the system size L via $e^l = L/\pi$. At the lowest order of the RG flow equation (3) in the main text, the boundary of the BKT transition corresponds to $\delta = 2g'$. Since $E_0 = E_3$ is equivalent to this condition, the BKT transition point is determined from the crossing point of these two energy levels. In our model, this corresponds to the crossing of the levels shown as the red dashed line and the blue solid line in Supplementary Fig. 4. In numerical calculations, we obtain the excitation energy of the energy level ($S^z = \pm 4, q = 0, P = 1$) by multiplying that of the level ($S^z = \pm 1, q = 0, P = 1$) by a factor of 16 to minimize a change in the field-theory parameters due to an increase in the total magnetization S^z in finite-size systems. We note that, even though we consider a non-Hermitian model here, the level spectroscopy method is applicable because the BKT phase boundary is entirely within the PT-unbroken region and the low-energy spectrum is thus equivalent to that of the sine-Gordon model as proved in the main text. We calculate the transition point for different system sizes (Supplementary Fig. 4(a)-(c)), and extrapolate it to the thermodynamic limit

to determine the BKT transition point (Supplementary Fig. 4(d)). Since $-\Delta = \cos(\pi/2K)$ for $h_s = \gamma = 0$ and the BKT transition occurs near $K = 2$, our analysis focuses on a region around $-\Delta = \cos(\pi/4) = 1/\sqrt{2}$.

The PT threshold is determined from the first coalescence point in the low-energy spectrum. To confirm that the identified point indeed represents an exceptional point of the spectrum, we plot the square of the energy difference $(\delta E)^2$ and test the square-root scaling of δE which appears when an exceptional point is formed by the coalescence of two eigenstates [1, 34] (see insets in Supplementary Fig. 4(a)-(c)). We then perform a linear fit to the $(\delta E)^2$-γ plot and identify the PT threshold γ_{PT} for different system sizes. Finally, we extrapolate it to the thermodynamic limit and determine the PT symmetry breaking point (Supplementary Fig. 4(d)).

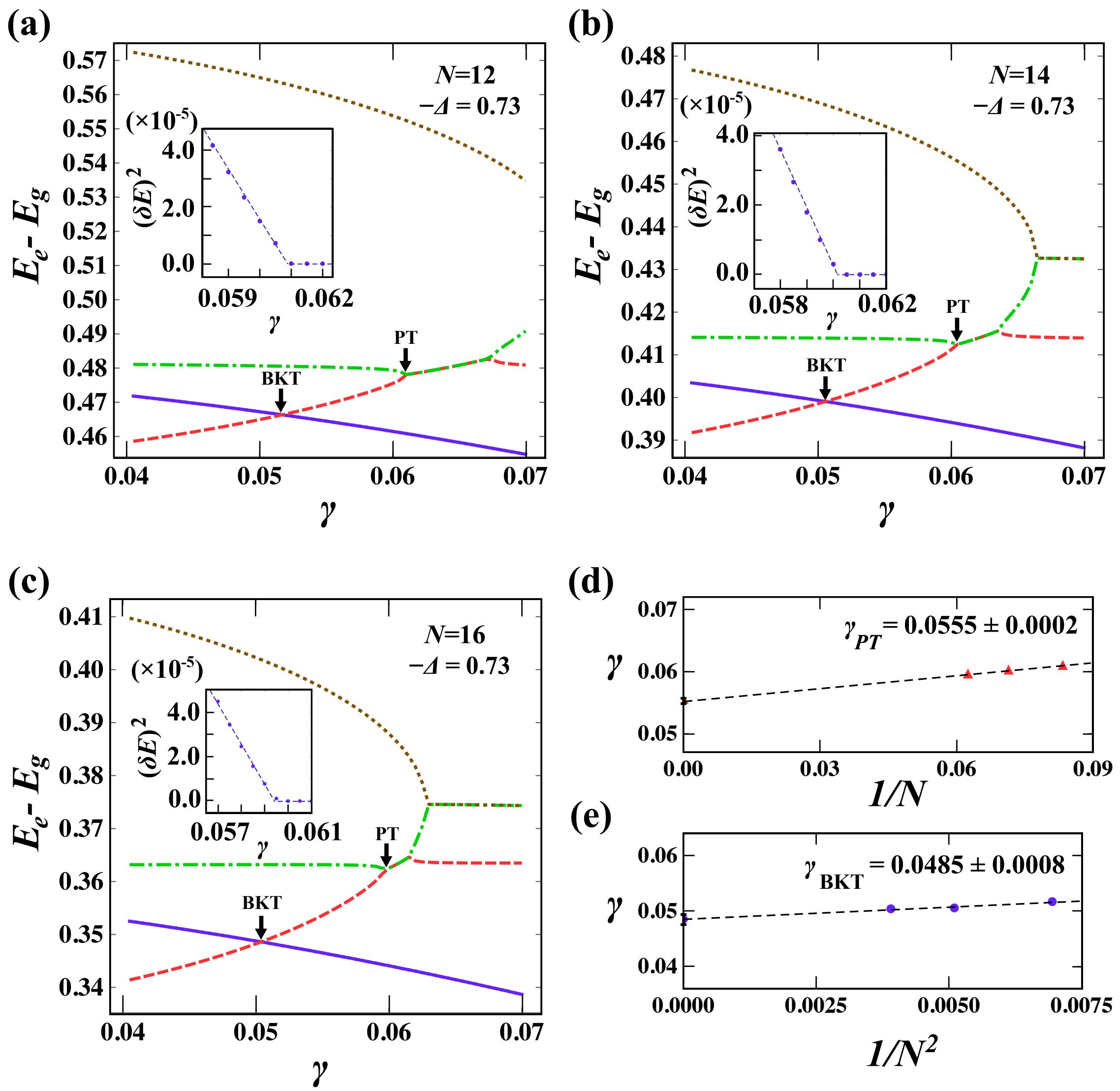

Supplementary Figure 4. **Exact finite-size spectra for different system sizes.** The spectra are plotted with the parameters $-\Delta = 0.73$ and $h_s = 0.1$ for different system sizes (a) $N = 12$, (b) $N = 14$, and (c) $N = 16$. Here the three lowest excited levels in the $(S^z = 0, q = 0, P = T = 1)$ sector (red, green, and yellow curves from the lowest), and the lowest excitation energy in the $(S^z = \pm 4, q = 0, P = 1)$ sector (blue curve) are plotted. The Berezinskii-Kosterlitz-Thouless (BKT) transition point corresponds to the crossing point of the two energy levels in $(S^z = 0, q = 0, P = T = 1)$ (red) and $(S^z = \pm 4, q = 0, P = 1)$ (blue). The PT transition point corresponds to the first coalescence point of two low-energy levels (e.g., red and green), which is confirmed to be an exceptional point of the spectrum by testing the square-root scaling of the energy difference δE between the two coalescing levels (inset). (d) The PT threshold (γ_{PT}) and (e) the BKT transition point (γ_{BKT}) are determined by extrapolating finite-size data to the thermodynamic limit.

Supplementary References

[1] Kato, T. *Perturbation Theory for Linear Operators.* (Springer, New York, 1966).

[2] Carmichael, H. *An Open System Approach to Quantum Optics.* (Springer, Berlin, 1993).

[3] Daley, A. J. Quantum trajectories and open many-body quantum systems. *Adv. Phys.* **63,** 77-149 (2014).

[4] Ashida, Y., Furukawa, S. & Ueda, M. Quantum critical behaviour influenced by measurement backaction in ultracold gases. *Phys. Rev. A* **94,** 053615 (2016).

[5] Facchi, P. & Pascazio, S. Quantum Zeno dynamics: mathematical and physical aspects. *J. Phys. A* **41,** 493001 (2008).

[6] Syassen, N. *et al.* Strong dissipation inhibits losses and induces correlations in cold molecular gases. *Science* **320,** 1329-1331 (2008).

[7] Garcia-Ripoll, J. J. *et al.* Dissipation-induced hard-core boson gas in an optical lattice *New J. Phys.* **11,** 013053 (2009).

[8] Reiter, F. and Sørensen, A. S. Effective operator formalism for open quantum systems. *Phys. Rev. A* **85,** 032111 (2012).

[9] Zhu, B. *et al.* Suppressing the loss of ultracold molecules via the continuous quantum Zeno effect. *Phys. Rev. Lett.* **112,** 070404 (2014).

[10] Giamarchi, T. *Quantum Physics in One Dimension.* (Oxford University Press, Oxford, 2004).

[11] Cazalilla, M. A. *et al.* One dimensional bosons: from condensed matter systems to ultracold gases. *Rev. Mod. Phys.* **83,** 1405-1466 (2011).

[12] Turlapov, A., Tonyushkin, A. & Sleator, T. Optical mask for laser-cooled atoms. *Phys. Rev. A* **68,** 023408 (2003).

[13] Oberthaler, M. K. *et al.* Dynamical diffraction of atomic matter waves by crystals of light. *Phys. Rev. A* **60,** 456–472 (1999).

[14] Johnson, K. S. *et al.* Localization of metastable atom beams with optical standing waves: nanolithography at the Heisenberg limit. *Science* **280,** 1583–1586 (1998).

[15] Stützle, R. *et al.* Observation of nonspreading wave packets in an imaginary potential. *Phys. Rev. Lett.* **95,** 110405 (2005).

[16] Fukuhara, T. *et al.* Quantum dynamics of a mobile spin impurity. *Nature Phys.* **9,** 235-241 (2013).

[17] Fukuhara, T. *et al.* Spatially resolved detection of a spin-entanglement wave in a Bose-Hubbard chain. *Phys. Rev. Lett.* **115,** 035302 (2015).

[18] Weitenberg, C. *et al.* Single-spin addressing in an atomic Mott insulator. *Nature* **471,** 319-324 (2011).

[19] Islam, R. *et al.* Measuring entanglement entropy in a quantum many-body system. *Nature* **528,** 77-83 (2015).

[20] Sherson, J. F. *et al.* Single-atom-resolved fluorescence imaging of an atomic Mott insulator. *Nature* **467,** 68-72 (2010).

[21] Endres, M. *et al.* Observation of correlated particle-hole pairs and string order in low-dimensional Mott Insulators. *Science* **334,** 200-203 (2011).

[22] Bloch, I., Dalibard, J. & Nascimbène, S. Quantum simulations with ultracold quantum gases. *Nature Phys.* **8,** 267-276 (2012).

[23] Bakr, W. S. *et al.* A quantum gas microscope for detecting single atoms in a Hubbard-regime optical lattice. *Nature* **462,** 74-77 (2009).

[24] Greiner, M. *et al.* Quantum phase transition from a superfluid to a Mott insulator in a gas of ultracold atoms. *Nature* **415,** 39-44 (2002).

[25] Endres, M. *et al.* The Higgs amplitude mode at the two-dimensional superfluid/Mott insulator transition. *Nature* **487,** 454-458 (2012).

[26] Hofferberth, S. *et al.* Probing quantum and thermal noise in an interacting many-body system. *Nature Phys.* **4,** 489-495 (2008).

[27] Sarchi, D., Carusotto, I., Wouters, M. & Savona, V. Coherent dynamics and parametric instabilities of microcavity polaritons in double-well systems. *Phys. Rev. B* **77,** 125324 (2008).

[28] Carusotto, I. & Ciuti, C. Spontaneous microcavity-polariton coherence across the parametric threshold: Quantum Monte Carlo studies. *Phys. Rev. B* **72,** 125335 (2005).

[29] Wouters, M. & Carusotto, I. Absence of long-range coherence in the parametric emission of photonic wires. *Phys. Rev. B* **74,** 245316 (2006).

[30] Gladilin, V. N., Ji, K. & Wouters, M. Spatial coherence of weakly interacting one-dimensional nonequilibrium bosonic quantum fluids. *Phys. Rev. A* **90,** 023615 (2014).

[31] Trotzky, S. *et al.* Probing the relaxation towards equilibrium in an isolated strongly correlated

1D Bose gas. *Nature Phys.* **8,** 325-330 (2012).

[32] Nomura, K. Correlation functions of the 2D sine-Gordon model. *J. Phys. A* **28,** 5451 (1995).

[33] Kitazawa, A., Nomura, K. & Okamoto, K. Phase diagram of $S = 1$ bond-alternating XXZ chains. *Phys. Rev. Lett.* **76,** 4038-4041 (1996).

[34] Heiss, W. D. & Steeb, W.-H. Avoided level crossings and Riemann sheet structure. *J. Math. Phys.* **32,** 3003-3007 (1991).
